# Reply to Claus Juergen Preusse

**DOI:** 10.1093/icvts/ivag055

**Published:** 2026-03-23

**Authors:** Fabrizio Settepani

**Affiliations:** Cardiac Surgery and Heart Transplant Unit, Cardio-Thoraco-Vascular Department, Niguarda Hospital, Milan 20162, Italy

We appreciate Dr Claus Juergen Preusse’s engagement with our recent publication and welcome the opportunity to clarify the methodological points raised.^1–2^

## USE OF P-VALUES VERSUS EFFECT SIZE AND CONFIDENCE INTERVALS

Our study was designed as a retrospective observational analysis with pairwise comparisons between each cardioplegic strategy (Custodiol and St. Thomas) and a predefined reference group (Celsior), rather than as a global 3-arm comparison. Accordingly, the statistical tests applied—Student’s *t*-test, χ^2^/Fisher exact test, Kaplan-Meier analysis with log-rank testing, and Cox proportional hazards models—were selected *a priori* and are routinely used in transplant outcomes research.^3^

We acknowledge the broader debate regarding *P*-values, as outlined in the ASA statement and in Ioannidis’ work. These statements caution against over-interpretation rather than appropriate use. Reporting *P*-values alongside effect sizes and confidence intervals, as performed in our Cox analyses, aligns with modern reporting standards and enhances interpretability. Recent cardioplegia and transplant literature similarly supports this approach to convey clinical uncertainty.^3^

## GROUP COMPARABILITY AND REPRESENTATIVENESS

Baseline demographic and procedural characteristics were comprehensively compared between each study group and the reference group. No clinically relevant imbalances were identified that would materially confound outcome assessment. Cox proportional hazards modelling provided additional adjustment for factors influencing survival, consistent with best practices in observational comparative research.

## DIFFERENCES IN CARDIOPLEGIA DELIVERY PROTOCOLS

Differences in cardioplegic administration reflect the intended clinical use of each solution. HTK-Custodiol is designed for single-dose delivery during prolonged cold ischaemia, whereas Celsior and St. Thomas cardioplegia are routinely administered with repeated dosing. Altering these protocols would have resulted in nonstandard or off-label use, thereby reducing real-world applicability. Our analysis, therefore, compares practical myocardial protection strategies as applied in contemporary heart transplantation.

## PRESENTATION OF CONFIDENCE INTERVALS IN SURVIVAL ANALYSIS

While graphical display of confidence intervals may aid visual interpretation, it is not required for analytical validity. Importantly, confidence intervals were provided in the numerical reporting of Kaplan-Meier estimates (eg, survival at 1, 5, and 12 years in the St. Thomas group was 81.5 ± 3.4%, 71.9 ± 4.1%, and 65.5 ± 5.2%, respectively), demonstrating no meaningful survival differences versus the reference group (*P* = .64).^2^

## CLINICAL RELEVANCE OF THE FINDINGS

We respectfully disagree with the assertion that the study lacks clinical relevance. Optimal myocardial protection in heart transplantation remains an area of active investigation, and long-term comparative data are limited. Despite the inherent limitations of retrospective analyses, our study provides meaningful long-term outcome data reflective of real-world practice.

Moreover, the manuscript underwent standard peer-to-peer review under the Editor’s supervision before acceptance, supporting its methodological soundness and clinical relevance.

In summary, our analytical approach was deliberate, appropriate to the study design, and consistent with established clinical research standards.
